# Expanding the understanding of local community assembly in adaptive radiations

**DOI:** 10.1002/ece3.908

**Published:** 2013-12-21

**Authors:** Katharina C Wollenberg, Michael Veith, Stefan Lötters

**Affiliations:** 1 Department of Biology School of Science, Engineering and Mathematics, Bethune-Cookman University640 Dr Mary McLeod Bethune Blvd, Daytona Beach, Florida, 32114; 2 Department of Biogeography, Trier UniversityUniversitätsring 15, 54296, Trier, Germany

**Keywords:** Adaptive radiation, body mass, community assembly, environmental filtering

## Abstract

Communities are thought to be assembled by two types of filters: by the environment relating to the fundamental niche and by biotic interactions relating to the realized niche. Both filters include parameters related to functional traits and their variation along environmental gradients. Here, we infer the general importance of environmental filtering of a functional trait determining local community assembly within insular adaptive radiations on the example of Caribbean *Anolis* lizards. We constructed maps for the probability of presence of *Anolis* ecomorphs (ecology-morphology-behavior specialists) on the Greater Antilles and overlaid these to estimate ecomorph community completeness (ECC) over the landscape. We then tested for differences in environmental parameter spaces among islands for real and cross-fitted ECC values to see whether the underlying assembly filters are deterministic (i.e., similar among islands). We then compared information-theoretic models of climatic and landscape parameters among Greater Antillean islands and inferred whether body mass as functional trait determines ECC. We found areas with high ECC to be strongly correlated with environmental filters, partly related to elevation. The environmental parameters influencing high ECC differed among islands. With the exception of the Jamaican twig ecomorph (which we suspect to be misclassified), smaller ecomorphs were more restricted to higher elevations than larger ones which might reflect filtering on the basis of differential physiological restrictions of ecomorphs. Our results in *Anolis* show that local community assembly within adaptive island radiations of animals can be determined by environmental filtering of functional traits, independently from species composition and realized environmental niche space.

## Introduction

The idea that community assembly is largely competition-based (Diamond 1975; Adams 2007) today is relativized by the identification of additional determining factors. Community assembly is now thought of being determined by two types of filters: by the environment relating to the fundamental niche and by biotic interactions relating to the realized niche (Weiher et al. 2011). Both filters act upon functional traits and their variation along environmental gradients (Uriarte et al. 2010; Weiher et al. 2011). Functional traits, such as leaf size, can determine species persistence in a local community (Ackerly 2003; Kraft and Ackerly 2010). Community assembly by functional traits (which is a deterministic process and thus predictable) is not necessarily associated with the phylogenetic relationships within the community, which in turn determine species composition (usually not being predictable, Fukami et al. 2005; Ingram and Shurin 2009; Uriarte et al. 2010; Weiher et al. 2011; Graham et al. 2012). Convergence of community functional traits across similar climatic parameters has, for example, been identified in South American tropical forests (Kraft et al. 2008), stream fishes of Europe and North America (Lamouroux et al. 2002), and bat assemblages across latitudinal gradients (Stevens et al. 2003), which suggests that niche-based assembly might be the norm (Garnier et al. 2007; Weiher et al. 2011).

**Figure 1:**
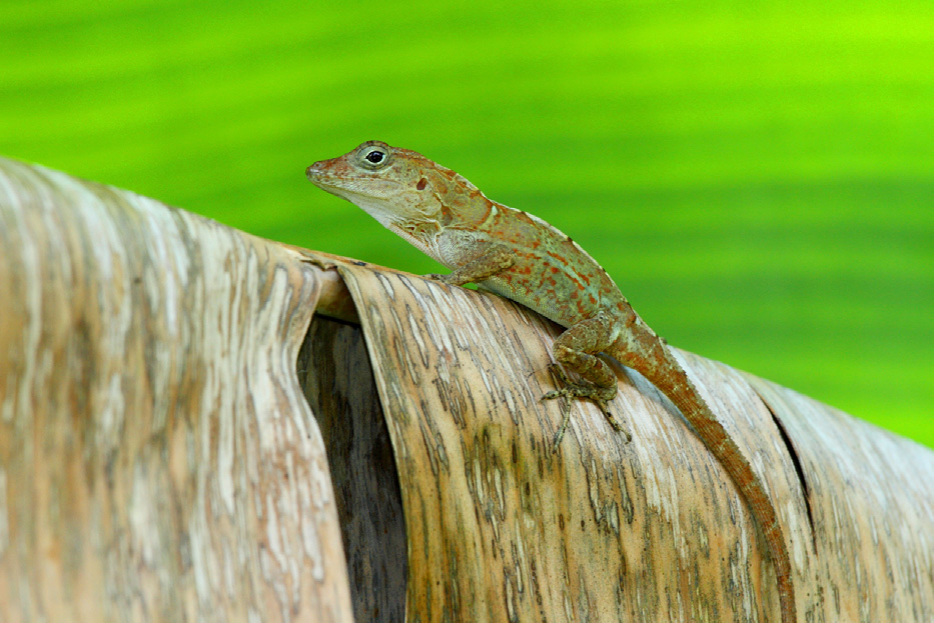
*Anolis cybotes*, a trunk-ground ecomorph from the Dominican Republic. Copyright Miguel A. Landestoy, 2010. Reproduced with permission.

While a large body of evidence shows that plant communities are clearly locally assembled by their functional traits (Ackerly 2003; Kraft et al. 2008), local community assembly in animals is understudied in this regard. Especially in adaptive animal radiations, the general consensus is that ecomorphological divergence arose by resource competition (a form of biological interaction filtering) which led to ecological speciation (Williams 1972; Streelman and Danley 2003; Parnel and Streelman 2011). Ecological speciation is defined as the evolution of reproductive isolation between populations resulting from ecologically based divergent natural selection (Schluter and Conte 2009). The deterministic nature of this process sometimes led to convergent evolution of similar ecomorph communities within the adaptive radiation (Losos et al. 1998; Losos and Ricklefs 2009). Beyond these general patterns of divergence influencing functional traits, local community assembly within such adaptive radiations has most commonly been investigated from the perspective of species distribution (Gillespie 2004; Esselstyn et al. 2011; Parnel and Streelman 2011; Lee et al. 2012; Algar et al. 2013). For example, speciation of spiders among Hawaiian Islands (Gillespie 2004) or Greater Antillean *Anolis* lizards (Losos et al. 1998; Algar et al. 2013; Fig. 1) leads to the presence of nonrandom parallel sets of ecomorphs, with nonoverlapping distributions of species belonging to the same ecomorph. In contrast, to date, it has not been assessed to which degree animal ecomorphs that arose through adaptive radiation are also locally assembled according to their functional traits and whether this is a deterministic process.

One such example can be found in the adaptive radiation of Greater Antillean *Anolis* lizards. Their high diversity is allocated to two factors (Losos 2009): convergent evolution of the same set of ecomorphs on each of the four islands (Williams 1972) and subsequent diversification within these ecomorphs (e.g., Glor et al. 2003; Glor and Warren 2011). Ecomorph evolution occurred, according to a widely accepted hypothesis (Williams 1972), by diversification due to interspecific competition into diverse structural microhabitats. This differential structural habitat use is accompanied by phenotypic and behavioral differences (Irschick and Losos 1998; Kolbe et al. 2012). On each Greater Antillean island, similar ecomorph sets are repeatedly found among distantly related clades, likely as a result of convergence (Losos et al. 1998).

As the utilized structural microhabitats exploited by all ecomorphs are generally present throughout the islands (Losos 2009), one should therefore expect “complete” ecomorph communities at each locality. Yet, this is not the case (Losos 2009) – but the underlying filters determining local ecomorph community assembly have not been comprehensively assessed to date (but see Haefner 1988). For example, the so-called trunk-ground and trunk-crown ecomorphs are present in most localities on all islands of the Greater Antilles (Losos 2009). This means that almost the complete island area is allopatrically subdivided by species belonging to the same ecomorph (e.g., Glor et al. 2003; Glor and Warren 2011; Algar et al. 2013). Other ecomorphs show more restricted distributions – a portion of the geographical range of an island is not used by any species belonging to these respective ecomorphs. It is therefore thinkable that factors additional to biotic interaction filters are driving the local coexistence of ecomorphs (Losos 2009).

One candidate variable is macrohabitat type and related microhabitat availability: For example, the twig anoles on Jamaica exclusively occur in lowland grasslands, which provide a reduced perch spectrum (Losos 2009). But twig anoles on other islands occur in different habitats and elevations (the twig anoles on Hispaniola occur only in the highlands, where grasslands are absent). Macrohabitat type (and related microhabitat availability) is therefore not likely to limit local ecomorph community completeness.

While the spatial distribution of a species within an ecomorph class is suggested to be limited by competition with other species of the same ecomorph class (Esselstyn et al. 2011; Glor and Warren 2011; Algar et al. 2013), the spatial distribution of all species in the same ecomorph class should not be limited by such competition (Losos 2009). Their characteristic differences in functional traits irrespective of species composition make Greater Antillean *Anolis* lizard ecomorphs an ideal model system to test whether environmental filters determine the distribution of ecomorphs and thus the local assembly of ecomorph communities within such an adaptive insular radiation.

Based on community assembly theory, in this paper, we test whether local presence of ecomorphs is correlated with broad scale environmental filters and whether this is due to ecomorph-specific differences in body mass potentially related to physiology. Body mass is a known functional trait in animals and has been found to explain metabolic rate variation in squamate reptiles (Andrews and Pough 1985; Gillooly et al. 2001).

(1) First, we infer whether environmental filters promote different levels of completeness in ecomorph community assembly (ecomorph community completeness, ECC). Since the ecomorph communities on the Greater Antillean Islands have convergently evolved four times, we also investigate if the niche spaces that determine high ECC on the different islands overlap. In this way, we infer whether convergent ecomorph communities also represent deterministic community assembly by convergence in occupied environmental niches.

(2) The high gradient of variation in climate and topographic structure on these islands could in theory produce animals with a differential thermal and hydric physiology (Losos 2009), some of which might have narrower climatic tolerances and therefore not be able to occur everywhere. To further investigate the possible relationship between ecomorph type, environment and physiology, we test for differences in the relationship between ecomorph occurrence and environment depending on ecomorph body mass. We expect to find body mass of ecomorphs influencing their environmental niches and thus community assembly.

## Methods

Six categories of Greater Antillean anole ecomorphs are currently known. Ecomorphs are defined as “species with the same structural habitat/niche, similar in morphology and behaviour, but not necessarily close phyletically” (Williams 1972). The morphological component includes body size (body mass), relative limb and tail length, number of lamellae on toe pad, and color (reviewed in Losos 2009). Definitions of ecomorph are adapted from Losos (2009): crown-giant (large species that inhabit the crowns of trees), trunk-crown (slender-bodied lizards that inhabit the upper portion and branches of a tree), trunk-ground (stocky-bodied lizards that inhabit the lower portion of a tree and are also frequently found on the ground), trunk (smaller lizards with flattened body and short limbs that are exclusively found on trunks), grass-bush (small, slender lizards with long tails and relatively long limbs that are found on shrubs and in grassy vegetation), and twig (small lizards that have extremely short limbs, elongated bodies, chameleon-like behavior, and are perching on small twigs).

On each island, more than one species per ecomorph can be present (see Losos 2009). We collected approximately 27,300 distribution records for all Greater Antillean *Anolis* species belonging to an ecomorph (in total 107 species, see afterword in Losos 2009 for a list of names) to construct Ecological Niche Models, ENMs (Franklin 2009) for each ecomorph per island. Georeferenced records were obtained from two sources (Herp.net and GBIF data portals, accessed in January and March 2012) and partially supplemented by records georeferenced manually (Data available from the Dryad digital repository: http://doi.org/10.5061/dryad.4b2g5). We aimed for 30 unique localities per species and were able to get this number for all species except *Anolis loysianus,* the Cuban trunk anole. Manual georeferencing had to be performed for many species on Puerto Rico, and only for records whose exact location (province, town, and km distance) was indicated on the museum record. Each species' distribution localities were checked by visualizing them in ArcGIS, and locality records that were obviously incorrect or outliers with respect to the species distribution were removed prior to analysis.

These translated into ∼11,000 unique locality records (i.e., localities where a species was present with more than one specimen were reduced to one coordinate for the respective species). In order to create ENMs, we used the widely used 19 bioclimatic layers of Busby (1991), obtained from WorldClim (Hijmans et al. 2005). ENMs and maps of ecomorph habitat suitability scores were computed with Maxent 3.3.3k (Phillips et al. 2006; Phillips and Dudík 2008). We employed Maxent under standard settings and created each 100 models (random seed) per ecomorph per island with 5000 background points. Estimated probability of presence at sites with “typical” conditions for each ecomorph was set to Τ = 0.5 (Elith et al. 2011). *Anolis* ecomorphs in their entirety occupy all parts of the Greater Antillean Islands without obvious range restrictions (and intraecomorph competition for distribution areas can be neglected since they were analyzed as pooled into ecomorphs). Furthermore, enough time has passed since their evolution during which each ecomorph in theory could have colonized all parts of these islands, validating the probability of presence of Τ = 0.5. ENMs and sampling data per ecomorph and island are deposited in the Dryad data repository (http://doi.org/10.5061/dryad.4b2g5)

Because we were not only interested in obtaining ENMs for each ecomorph on its own island, but also in exploring these presence probability scores fitted to the other islands, the complete Greater Antilles were used as the background area. Twenty-five percent of records were each randomly used for model testing via the area under the receiver operating characteristic curve (AUC), as implemented in Maxent. Most AUC values ranged 0.8–0.9 and few ranged 0.7–0.8, thus suggesting “good” and “useful” discrimination ability, respectively. Only Cuban crown-giant and twig anole models had AUC > 0.6, suggesting poor discrimination ability but were still better than expected by a random model. With regard to hypothesis (1), we subsequently performed the following analyses: The resulting ENMs per ecomorph per island and those projected to the complete Greater Antilles were imported into ArcGIS10 v.10 (ESRI, Redlands, CA). Scaling was equal between ecomorphs (values between 0 and 1) and then summed up to obtain a spatial representation of our main variable of interest, ECC. We calculated weighted sums of ENMs of all ecomorphs per each island and for the values per ecomorph fitted to all other islands in ArcMAP. ECC values range from 0 to 6 because 6 is the maximal number of ecomorphs that evolved on any of the Greater Antillean Islands.

We assessed similarity between ENMs of the whole Greater Antilles (background), calculated on the basis of each island (data); niche overlap between the models for each ecomorph and for ECC was calculated as modified Schoener's D (Schoener 1968), as implemented in ENMtools (Warren et al. 2010). We then inferred whether ECC was correlated with elevation on each island. We ran correlation analyses between each island's ECC and elevation using ENMtools. As ENMtools does not calculate *P*-values, we manually calculated them using a reduced-size dataset. The obtained *P*-values were added to the correlation statistics obtained via ENMtools. We extracted point values for elevation, bioclimatic information, and ECC from each grid cell. Subsequently, we applied principal component analyses (PCAs) on the bioclimatic variables in order to reduce their dimensionality (with STATISTICA 7.0, StatSoft, Tulsa, OK). From this PCA, five bioclimatic principal components (BioPCs) were retained based on the criterion of Eigenvalue >1, see Table S1, S2. Because of the large number of point values (∼11,000), correlations will almost always yield high *P*-values due to population effects. In order to both reduce the number of data points as also the amount of redundancy in the selected points, we performed a cluster analysis using *k*-means = 100 in the software PAST 2.17b (O. Hammer, Geological Museum of Copenhagen) on the extracted BioPCs to group points according to their bioclimatic similarity. From these points, we randomly selected each 1 or 10 points from each cluster, using the R package *plyr* v.1.8 (Wickham 2012), yielding two datasets with 1000 and 100 points in total, respectively. The correlation between ECC and elevation for the 1000 points dataset was repeated for Puerto Rico and Jamaica, and for the 100 points dataset representing Cuba and Hispaniola (in order to yield similar numbers of points with respect to different sizes of these island pairs).

Ecomorph community completeness values could in theory differ between Jamaica and the other islands because two ecomorphs are missing on Jamaica (one ecomorph is missing on Puerto Rico). To check whether an ECC-elevation correlation would be mainly caused by ecomorphs additionally present on Puerto Rico, Cuba, and Hispaniola, we repeated the correlation analyses for these three islands without these additional ecomorphs (Grass-bush on Puerto Rico and Grass-bush and Trunk on Cuba and Hispaniola) to see whether this would render the correlation insignificant.

In order to identify the best model explaining ECC on each island (from the predictors elevation and BioPCs), we used an information-theoretic framework, derived from Kullback–Leibler information (Burnham and Anderson 2002) that measures the strength of evidence for all competing models from a meaningful candidate set for the full dataset (∼11,000 data points). We constructed models as follows: (1) a global model including elevation and all five BioPCs; (2) elevation only; (3) all BioPCs but not elevation; (4) elevation-correlated BioPCs; (5) elevation-independent BioPCs. Not all BioPCs were congruently elevation-dependent or-independent among all islands; models (4) and (5) therefore were composed of unique variable sets per island. For Jamaica, all BioPCs were correlated with elevation. Instead of competing elevation-dependent (4) and-independent models (5), we therefore used models (1–3) and (6–10), the latter set containing each BioPC separately (BioPC1–BioPC5). We ranked the models according to the Akaike Information Criterion corrected for finite sample sizes (AICc). Model uncertainty was assessed by comparing ΔAICc values and Akaike weights (Burnham and Anderson 2002) in which the lowest values for ΔAICc indicate the most parsimonious models of the set. We considered models with Akaike weight ≥0.9 as single most parsimonious model. If the ΔAICc between the best-supported and the next suitable model were <3, we used model averaging (Burnham and Anderson 2002) to calculate regression coefficients, standard errors, and 95% confidence intervals for all variables. Model selection was carried out with the AICcmodavg package v. 1.27 (Mazerolle 2013) and model averaging with the MuMIn package v. 1.9.0 (Bartón 2013) in R. To find out whether the difference in best-supported explanatory models among islands is reflected in numeric differences of bioclimatic variables between islands, we also compared BiocPCs between islands using Kruskal–Wallis analyses of variance (ANOVA).

Ecomorphs are defined based on a set of morphological, ecological, and behavioral characteristics. They do, however, clearly differ in their average body mass (according to Beuttell and Losos 1999). Differences in body mass are related to differences in metabolism and thus potentially physiological tolerances. In order to approach hypothesis (2), we therefore tested for correlation between ecomorph occurrence and elevation depending on ecomorph body mass. We categorized ecomorphs according to their body mass (taken from Beuttell and Losos 1999) as twig < grass-bush < trunk < trunk-crown < trunk-ground < crown-giant. While trunk-crown and trunk-ground anoles score similarly in terms of snout to vent length (SVL) and overall size, trunk-ground anoles are significantly larger in mass than trunk-crown anoles (Beuttell and Losos 1999). We argue that physiological constraints would be more related to body mass than size and therefore base our classification on body mass. Spearman *r* values obtained for each ecomorph for the correlation between elevation and habitat suitability of ecomorph occurrence were then correlated with the categorized ecomorph body mass. We found the Jamaican twig anole to present an outlier data point and repeated the analyses after its exclusion (see also Discussion).

## Results

### Environmental filters for ECC

**Figure 2:**
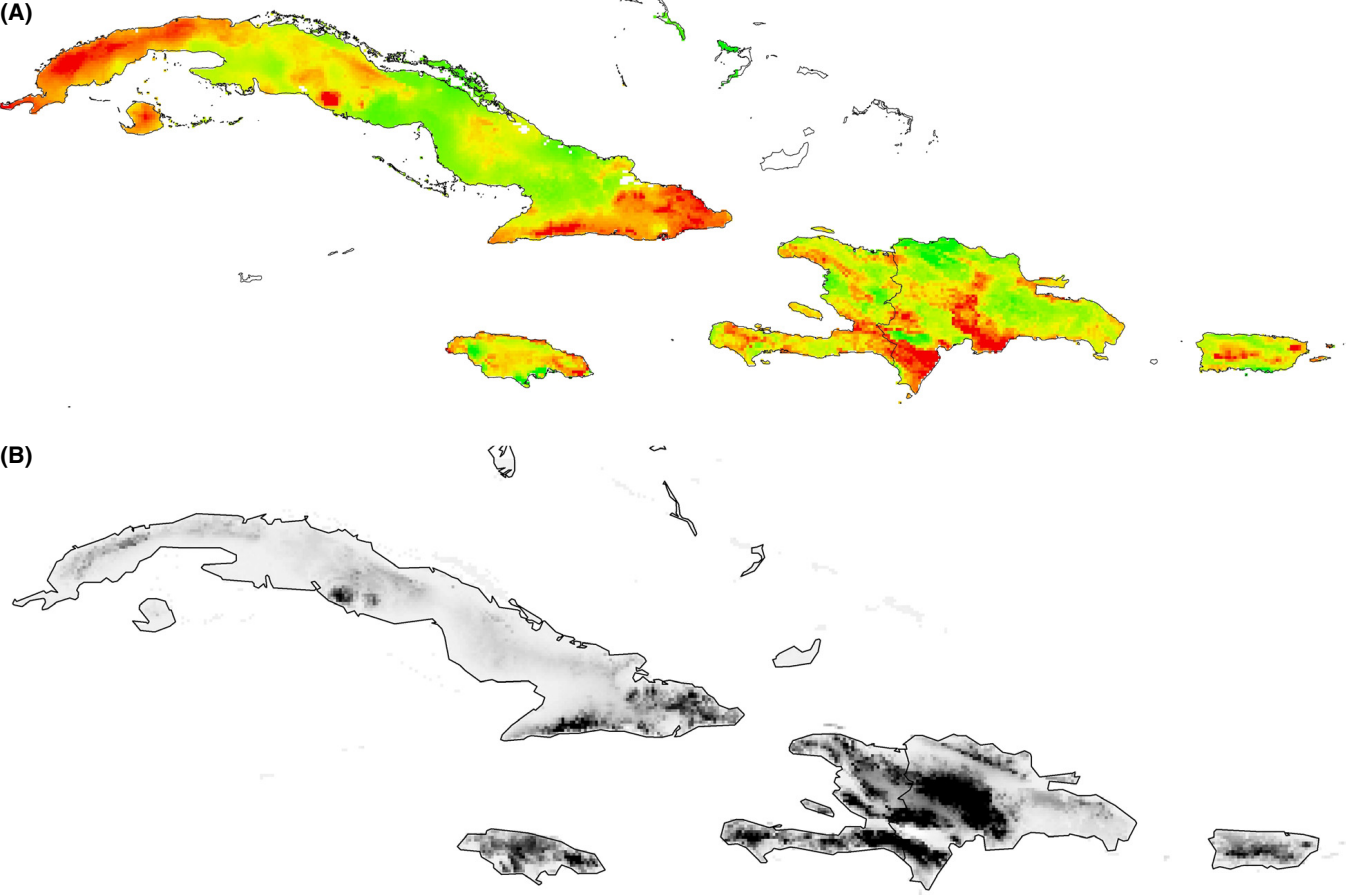
(A) Ecomorph community completeness for each island (red, dark – high, green, light – low); (B) topographical map of the Greater Antilles (dark – high, light – low).

We found ECC values on Hispaniola, Cuba, and Puerto Rico to almost perfectly correspond to the topographical map of the Greater Antilles, with an additional area of high ECC on the north-western tip of Cuba. In contrast, ECC on Jamaica was distributed almost equally across the island, reaching far beyond the island's central mountain range (Fig. 2A and B).

Niche overlap between models of ECC for the Greater Antilles, based on calculations for each island and cross-fitted to the other islands, revealed that niches for the calculated ECCs differ between most of the models (Table 1, see also Fig. S1). By default, the used similarity index (Schoener's D) will be around 0.5 when habitat suitability of two models largely overlaps at locations with intermediate scores (i.e., show no locations with clear similarities or dissimilarities). In our dataset, the only higher values of niche overlap were found between models computed for the Greater Antilles on the basis of data from Hispaniola and on the basis of data from Cuba (D = 0.629). In contrast, ENMs calculated on the basis of Hispaniola and Jamaica and on the basis of Cuba and Jamaica were the most different from each other. Models for the Greater Antilles calculated on the basis of Jamaica and Puerto Rico and on the basis of Puerto Rico and Hispaniola, or Puerto Rico and Cuba were neither highly similar nor highly dissimilar. In terms of single ecomorphs, the niches cross-fitted on basis of the crown-giant ecomorphs were highly similar among islands, where the niches cross-fitted for the twig ecomorphs were largely dissimilar (Table 1).

**Table 1:** Similarity between climatic niches for models based on each island, for each ecomorph and for ecomorph community completeness (ECC) using Schoener's D.

	ECC	Crown-giant	Trunk-crown	Trunk-ground	Trunk	Grass-bush	Twig
Hispaniola/Cuba	0.629	0.77	0.77	0.73	0.71	0.66	0.28
Hispaniola/Puerto Rico	0.524	0.71	0.73	0.75	Ecomorph absent	0.70	0.34
Hispaniola/Jamaica	0.205	0.82	0.59	0.59	Ecomorph absent	Ecomorph absent	0.26
Cuba/Puerto Rico	0.457	0.74	0.74	0.75	Ecomorph absent	0.67	0.38
Cuba/Jamaica	0.284	0.83	0.63	0.62	Ecomorph absent	Ecomorph absent	0.86
Puerto Rico/Jamaica	0.425	0.71	0.70	0.67	Ecomorph absent	Ecomorph absent	0.34

**Figure 3:**
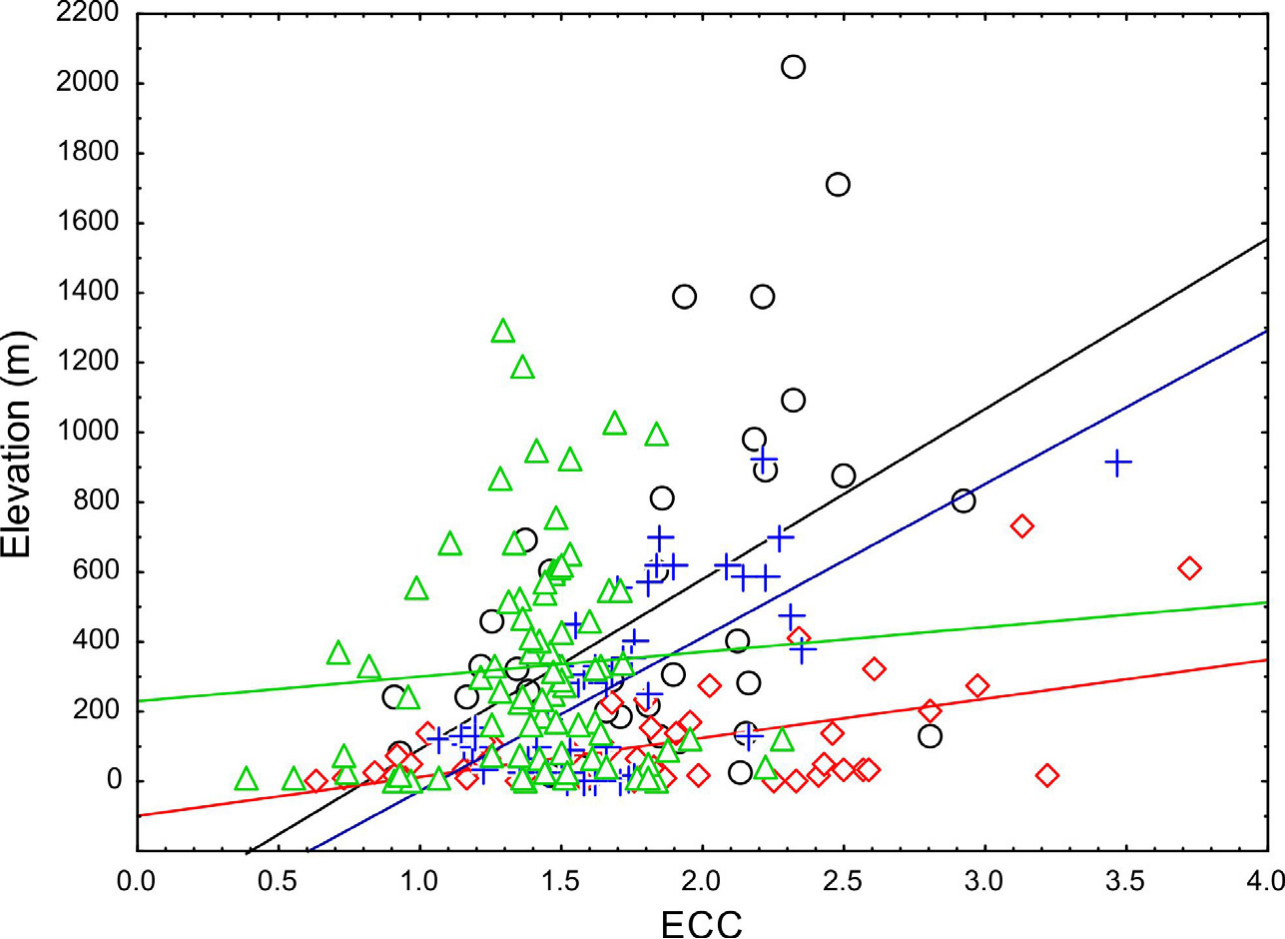
Correlation between ecomorph community completeness and elevation computed on reduced dataset of 1000 points for Puerto Rico and Jamaica, and for 100 points representing Cuba and Hispaniola: Hispaniola – black dots, Cuba – red diamonds, Puerto Rico – blue crosses, Jamaica – green triangles.

In light of the apparent connection between high ECC values and the topographical relief of the Greater Antilles, we correlated elevation with ECC on the basis of the ENMs and on the basis of extracted grid point values. Both model-based correlation as well as regular correlation on the extracted point dataset revealed that Puerto Rico and Hispaniola had the highest (significant positive) correlation coefficients between ECC and elevation, whereas the coefficient was lower but significant positive for Cuba and lowest as well as nonsignificant for Jamaica (Table 2, Fig. 3). In contrast, ECC in Jamaica peaked at mid-elevations (Table 2, Fig. 3). We tested whether the removal of ecomorphs that are not present on Jamaica (Grass-bush on Puerto Rico and Grass-bush and Trunk on Cuba and Hispaniola) would yield the ECC-elevation correlation insignificant, thus explaining the absence of a significant ECC-environment correlation on Jamaica. The correlation coefficient and error probabilities for this set of comparisons were as follows: Hispaniola (*r* = 0.648, *P* = 0.00001), Cuba (*r* = 0.406, *P* = 0.0038), and Puerto Rico (*r* = 0.739, *P* = 0.00001), so this possible explanation for the difference of Jamaica (= missing two ecomorphs) can be rejected.

**Table 2:** Correlation between elevation and ecomorph community completeness (ECC) on each of the Greater Antillean Islands.

Elevation	ECC Hispaniola	ECC Cuba	ECC Puerto Rico	ECC Jamaica
*R*	0.5403	0.3574	0.6163	0.0947
Slope	0.0023	289.499	394.119	0.0001
Intercept	1.5268	−118.515	−377.872	1.403
*r* (reduced dataset)	0.5367	0.4619	0.7069	0.076
Exact *P* (reduced dataset)	**0.00006**	**0.004**	**0.00000001**	0.5028

From top to bottom: Pearson correlation coefficient (*R*), slope, intercept, Spearman *r*,*P* derived from Spearman *r*.

Significant correlations are in bold.

We then performed AICc-based model selection to identify the combination of predictor variables best explaining ECC on each island and to investigate whether these models differ between Jamaica and the other islands. For Hispaniola and Cuba, the global model (including all bioclimatic principal components, BioPCs, and elevation) was the single most parsimonious one (Table 3). For Puerto Rico, the global model also fit best but with an Akaike weight of 0.89 and with a ΔAIC of 4.18 to the next best-fitting model. In contrast, the global model received a relatively lower Akaike weight for Jamaica (0.3336), where the best-fitting model was the one with only BioPCs, excluding elevation (0.6664). Model averaging revealed that three BioPCs were the best explanatory variables for ECC on Jamaica, with elevation being a nonsignificant predictor (Table 4). This means that community completeness of Jamaican ecomorphs is determined solely by bioclimatic filters (comprised of variables related to temperature range and precipitation, see Table S1). A Kruskal–Wallis test for differences in bioclimatic niche dimensions among Jamaica versus the other islands found significant differences in all BioPCs except the one with highest Eigenvalue.

**Table 3:** AICc-based ranking of models explaining ecomorph community completeness (ECC) for *Anolis* ecomorphs on the Greater Antillean Islands. For each model: ΔAICc (upper value, in italics); Akaike weights (lower value). For full results of AICc-based model selection, see Table S4. Elevation-correlated and-independent BioPCs are listed in parentheses for each island.

Model	Hispaniola	Cuba	Puerto Rico	Jamaica
1 “Global model”: Elevation + BioPC1–5	**0**	**0**	**0**	1.3835
	**1**	**1**	**0.89**	0.3336
2 Elevation only	1044.25	5463.44	112.77	104.1222
	0	0	0	0
3 All BioPCs-Elevation	66.98	676.92	4.18	**0**
	0	0	0.11	**0.6664**
4 Elevation-correlated BioPCs	(1)	(1, 5)	(1, 3, 4)	(1, 2, 3, 4, 5)
	842.67	3989.23	20.08	N/A
	0	0	0	
5 Elevation-independent BioPCs	(2, 3, 4, 5)	(2, 3, 4)	(2, 5)	
	1196.52	4101.36	322.48	N/A
	0	0	0	N/A
6–10 Each BioPC separately	N/A	N/A	N/A	47.341
				−106.046
				0

AICc, Akaike information criterion corrected for finite sample sizes; BioPC, bioclimatic principal components.

Best-supported models are marked bold.

**Table 4:** Model-averaged coefficients for Jamaica showing high relative importance for BioPC2, BioPC3, and BioPC5 in explaining ecomorph community completeness.

Variable	Estimate	Std. error	*Z* value	Exact *P* (>|*z*|)	Relative variable importance
(Intercept)	3.693e-01	1.548e-01	2.380	**0.0173**	N/A
Elevation	−1.487e-04	8.297e-05	1.789	0.0736	0.55
BioPC1	4.573e-02	2.447e-02	1.865	0.0621	0.58
BioPC2	2.233e-01	2.587e-02	8.612	**<2e-16**	1.00
BioPC3	4.871e-02	1.278e-02	3.804	**0.0001**	1.00
BioPC4	4.556e-02	5.616e-02	0.809	0.4183	0.29
BioPC5	3.696e-01	5.355e-02	6.887	**<2e-16**	1.00

BioPC, bioclimatic principal components.

Significant predictor variables are marked in bold.

### Influence of ecomorph body mass on ECC

**Figure 4:**
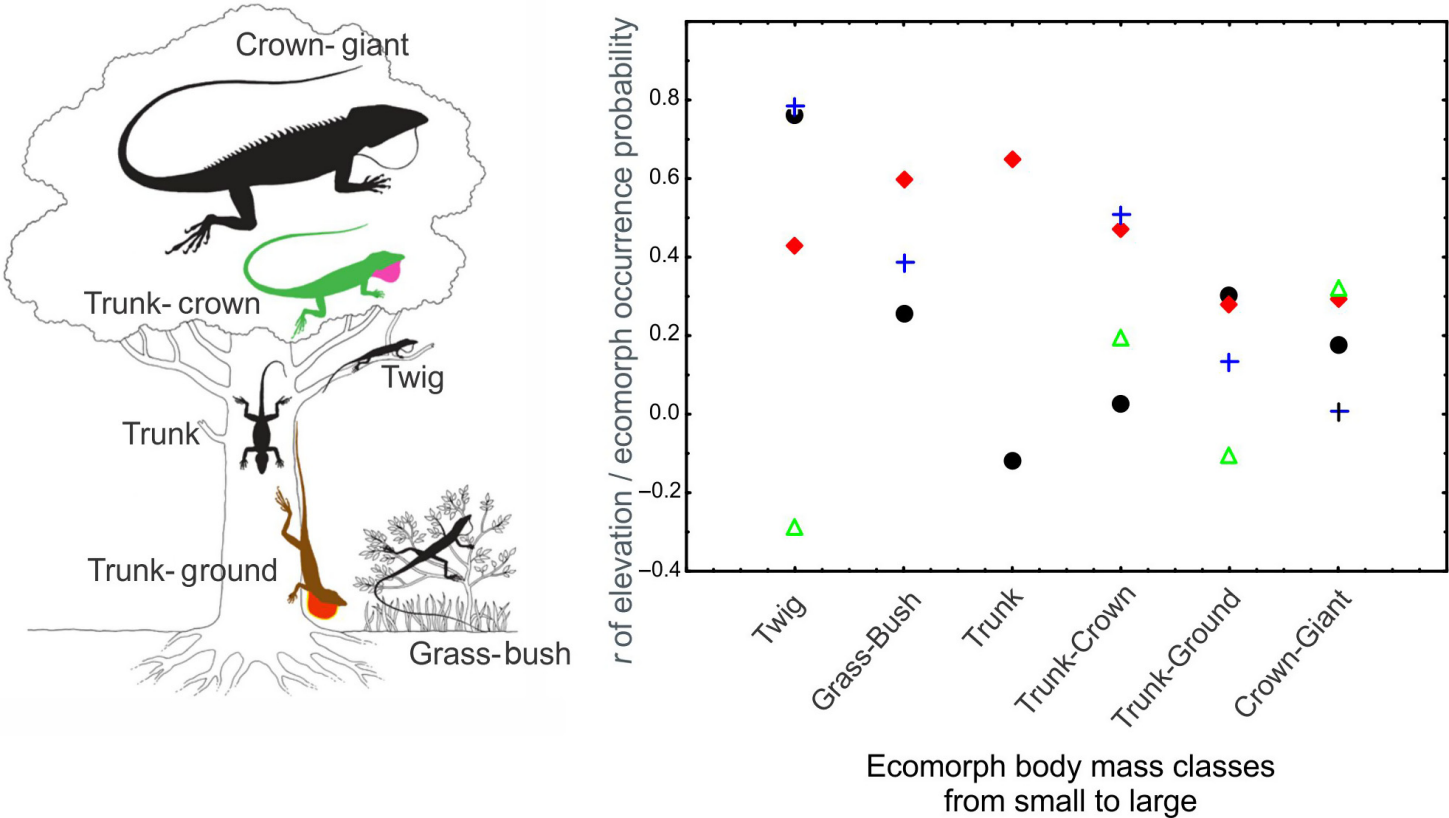
Left: Classical scheme of the *Anolis* ecomorphs (from Losos 2009; after Williams 1983). Right: negative correlation between elevational constraint and body mass classes of *Anolis* ecomorphs (*r* = −0.5826 and *P* = 0.007). Hispaniola – black dots, Cuba – red diamonds, Puerto Rico – blue crosses, Jamaica – green triangles.

From the full dataset (∼11,000 data points), we inferred the strength of correlation between habitat suitability obtained from ENMs for each ecomorph per island and elevation to see whether ecomorphs are restricted to high elevations (high *r*, elevational constraint) or not (low *r*). We then used this correlation coefficient to infer the relationship between the body mass classes of ecomorphs and elevational constraint. The Jamaican twig anole (only one species, *Anolis valencienni*) presented as an outlier, and we strongly believed the species to be misclassified in the twig ecomorph category (for rationale, see Discussion). We therefore excluded it which rendered the correlation highly significant (*r* = −0.5826 *P* = 0.007, Fig. 4).

## Discussion

### Community assembly filtering of Greater Antillean *Anolis* ecomorphs

In Greater Antillean anoles, ecological speciation leads to convergent evolution of similar communities of ecomorphs. In light of modern niche and community assembly theory (e.g., Holt 2009; Weiher et al. 2011), this refers to biotic interaction filtering. However, these filters cannot explain local ecomorph community assembly. Environmental filters have previously been found to be important in species-based processes within adaptive radiations (Soberón and Nakamura 2009; Ricklefs and Jenkins 2011; Wiens 2011), including *Anolis* (Knouft et al. 2006; Warren et al. 2008), but are yet understudied regarding their influence on functional traits and consequences for community assembly. On the basis of our results, we here advocate that environmental filtering on environmental niche axes explains the local community assembly of ecomorphs within this adaptive radiation. We inferred the effect of elevation and climate (which produce environmental niches) on local *Anolis* community assembly and identified a patchy distribution of areas where the complete community of ecomorphs is present on Hispaniola, Cuba, and Puerto Rico. This result that had been only corroborated by anecdotal evidence so far (see Losos 2009). In contrast, on Jamaica, almost the whole island was found suited for the complete set of ecomorphs.

As ecomorph communities have convergently evolved four times (Losos 1992; Losos et al. 1998), we also tested whether areas of ECC are mirrored by convergence in environmental niches, which would hint at deterministic community assembly. Attempts to quantify niche overlap of Greater Antillean anole ecomorph communities have been made before: Haefner (1988) used a null model approach to test whether niche shifts in spatially separated *Anolis* communities occurred within localities in Puerto Rico and Jamaica, but could not obtain a clear signal from their data. In our more comprehensive study involving all ecomorphs on all islands, we did not find models of ECC among any pairs of islands to be similar, with the exception of a slightly higher than random similarity between Hispaniola and Cuba. In the case of Jamaica versus Hispaniola and Jamaica versus Cuba, we found environmental niches to be even highly dissimilar. In contrast, on Hispaniola, Cuba, and Puerto, Rico ECC was highest in montane areas, and our data were explained best by the global model including elevation and all climatic variables, as opposed to elevation only. This means that ECC is determined by qualitatively similar, but quantitatively different bioclimatic parameters on all islands. Local community structure is known to be influenced by differential regional and historical processes (Ricklefs 1987). But given the fourfold convergence of anole ecomorph communities among islands and their strong spatial dependence on environmental filters (being elevation-related except on Jamaica), it is surprising that we found these to be associated with such strong quantitative differences in climate. On Jamaica, ECC is not following this pattern but is instead solely related to climatic variation. The missing correlation between ECC and elevation is likely not the result of the absence of two ecomorphs on this island, as we confirmed by repeating our analysis with an identical ecomorph set on all islands. We then identified several climatic parameters in Jamaica to be strikingly different to the other Greater Antillean islands, with daily and annual temperature ranges being much lower and elevation-independent, followed by local ECC equally mirroring this pattern. This incongruence in correlation results between Jamaica and the other Greater Antillean islands in the distribution of ECC and climate further supports the hypothesis that ECC is closely related to the bioclimatic component of the environment (with elevation dependency varying among locations).

### Body mass as a functional trait driving community assembly

The strong correlation between environmental niches and ECC leads to the question, why only some ecomorphs seem restricted to certain climatic parameter ranges (related to elevation on Puerto Rico, Hispaniola, and Cuba), eventually producing the observed local patchiness in ECC. For example, crown-giant anoles had large distribution areas with high similarity of climatic parameters among islands, whereas twig ecomorphs had restricted distributions and high niche dissimilarity among islands (exceptions can be found on Cuba and Jamaica). Elevational turnover in species composition has long been studied (e.g., Merriam 1894), as well as elevational turnover in species richness patterns (Diamond 1975; Wiens et al. 2007; Wollenberg et al. 2008) or endemism (Wollenberg et al. 2008). Evidence for certain ecomorphs being restricted to certain regions, independently of their phylogenetic origin, has been postulated for tree frogs (Wiens 2011).

In this paper, we identified a significant correlation between environmental filtering of *Anolis* ecomorph community assembly and ecomorph body mass as a functional trait: Smaller ecomorphs are restricted to higher elevations and corresponding climate. Larger (in terms of body mass) ecomorphs are found toward colder environments, potentially due to physiological restrictions of smaller ecomorphs in lower elevations (or, conversely, of larger ecomorphs in higher elevations). The ecomorph body mass–elevation relationship can also explain ECC patterns in Jamaica being different from the other islands due to the island's differential climate. The Jamaican twig ecomorph, which is distributed independently from elevation, consists of solely one species, *A. valencienni*. While similar in appearance to other twig anole species, the only available quantitative study (Williams 1983) categorized this species differently from the other twig ecomorphs; Due to its much larger body mass, and its perching habits being high up in the trees instead of low on the ground, *A. valencienni* was proposed to be a “giant twig” ecomorph as opposed to a dwarf twig like the other species in the ecomorph. The elevation-independent distribution of *A. valencienni* (presumably due to its larger body mass) could therefore cause the differential pattern in ECC and validates its exclusion from the twig category in our analysis. Lastly, we would like to point out that other functional traits that could co-vary with body mass and that we have not tested in this paper might underlie the found body size-elevation correlation. While body mass is a known functional trait in animals and has been found to explain metabolic rate variation in squamate reptiles (Andrews and Pough 1985; Gillooly et al. 2001), the exact relationship between body mass and physiological tolerances is still understudied in most *Anolis* species and warrants further study.

## Conclusions and Implications

Our results suggest that next to biotic interactive partitioning leading to the convergent evolution of a full set of *Anolis* ecomorphs on each island, environmental filters are a strong determinant of local ecomorph community assembly. Complete communities are restricted to areas defined by environmental niches that are suitable to the constraints of all ecomorphs' phenotypes. As the climatic parameter space defining high ECC largely differs between islands, this shows that local, qualitatively identical environmental filters determine local community assembly. This is potentially caused by ecomorph-specific physiological constraints that become evident even within quantitatively different environmental niche spaces. In conclusion, our results in *Anolis* expand the understanding of community assembly within insular adaptive radiations of animals. The current consensus is that biotic filters are of importance for their evolution, while environmental filters mostly influence species composition. Here, we could show that environmental filtering of functional traits independently from species composition is a major determinant for local community assembly, and future research should be directed toward investigating the importance of these filters in other adaptive animal radiations.

## Supporting Information

Figure S1 Ecomorphological community completeness in anoles for each Greater Antillean Island (green–low, red–high).

**Table S1.** Results of principal component analyses for the bioclimatic variables of the Greater Antilles (in Varimaxraw rotated coordinate system) by factor loadings (marked loadings are > 0.7). **Table S2.** Results of principal component analyses for the bioclimatic variables of the Greater Antilles (in Varimaxraw rotated coordinate system) by Eigenvalues. **Table S3.** Differences between Jamaica and other islands according to Kruskal–Wallis analyses of variance (ANOVA). **Table S4.** Complete results table of AICc-based model selection for ecomorph community completeness.
