# Supplementary material for: Expanding the understanding of local community assembly in adaptive radiations
Source: Ecol Evol. 2013 Dec 21;4(2):174–85. doi: 10.1002/ece3.908 (PMC3925381; doi:10.1002/ece3.908)

**Figure S1**. Ecomorphological Community Completeness (ECC) in anoles for each Greater Antillean Island (green – low, red – high). The ECC for each island computed on the basis of its own data is framed in red, remaining maps show ECCs extrapolated from conditions that are determining ECC on the other islands. Abbreviations are: HIS – Hispaniola, CU – Cuba, PR – Puerto Rico, JA – Jamaica. Not to scale.


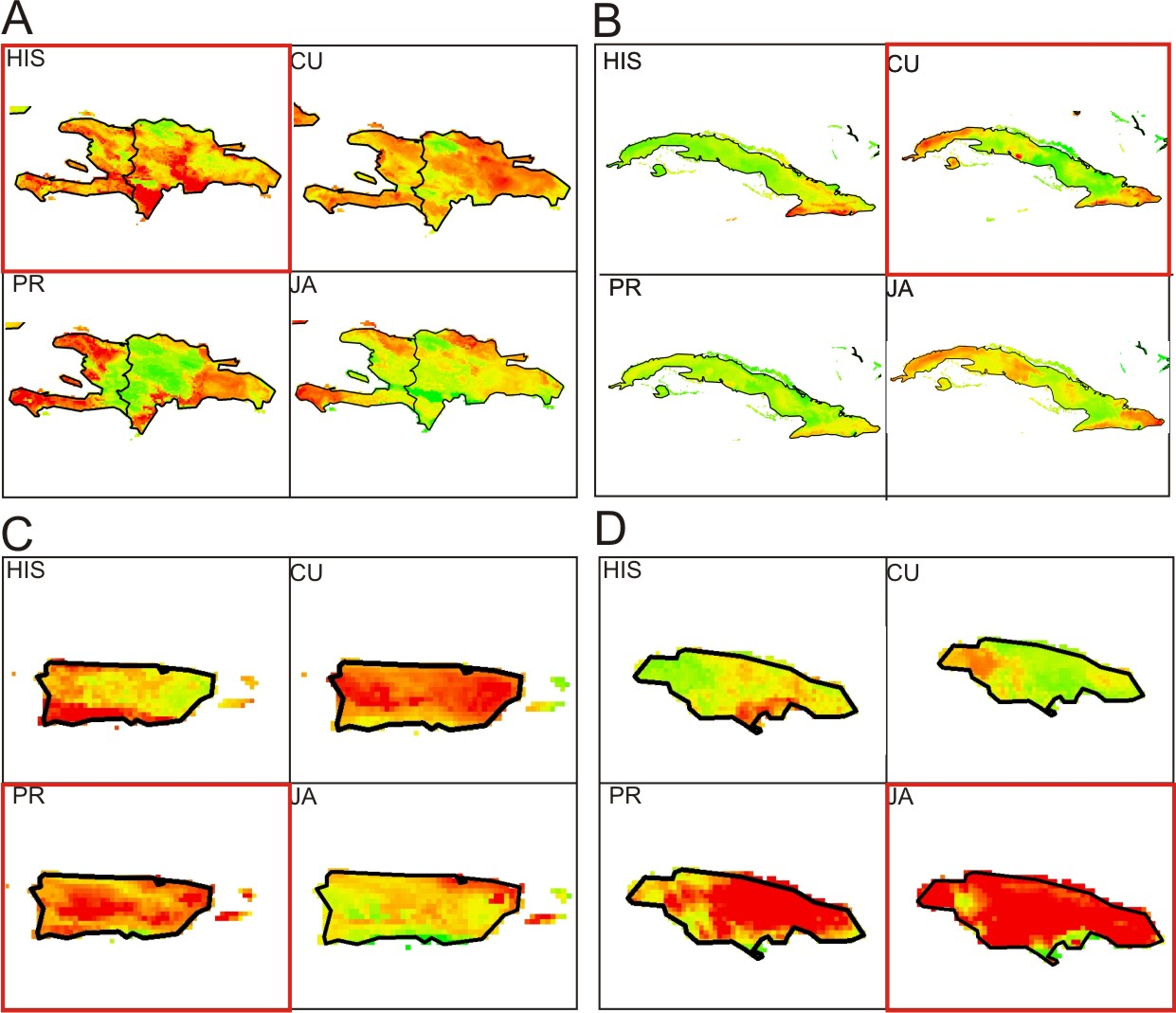

Supplement: Figure S1 — Ecomorphological community completeness in anoles for each Greater Antillean Island (green–low, red–high). [file ece30004-0174-sd1.docx]
