# Supplementary material for: Expanding the understanding of local community assembly in adaptive radiations
Source: Ecol Evol. 2013 Dec 21;4(2):174–85. doi: 10.1002/ece3.908 (PMC3925381; doi:10.1002/ece3.908)
Supplement: Supplementary file 2 — Table S1. Results of principal component analyses for the bioclimatic variables of the Greater Antilles (in Varimaxraw rotated coordinate system) by factor loadings (marked loadings are > 0.7). Table S2. Results of principal component analyses for the bioclimatic variables of the Greater Antilles (in Varimaxraw rotated coordinate system) by Eigenvalues. Table S3. Differences between Jamaica and other islands according to Kruskal–Wallis analyses of variance (ANOVA). Table S4. Complete results table of AICc-based model selection for ecomorph community completeness. [file ece30004-0174-sd2.docx]

**Supporting Information**

To the manuscript

**Expanding the understanding of local community assembly in adaptive radiations**

*Katharina C. Wollenberg, Michael Veith, Stefan Lötters*

Contains:

4 Supplementary Tables

1 Supplementary Figure

**Table S1.** Results of PCA for the bioclimatic variables of the Greater Antilles (in Varimax-raw rotated coordinate system) by factor loadings (marked loadings are > 0.7).

|  | **BioPC1** | **BioPC2** | **BioPC3** | **BioPC4** | **BioPC5** |
| --- | --- | --- | --- | --- | --- |
| **Bio1** | **0.980** | -0.076 | -0.134 | 0.111 | 0.039 |
| **Bio2** | -0.178 | -0.252 | -0.115 | -0.256 | **-0.908** |
| **Bio3** | -0.163 | 0.198 | -0.033 | **-0.928** | -0.238 |
| **Bio4** | 0.130 | -0.251 | 0.014 | **0.923** | -0.235 |
| **Bio5** | **0.894** | -0.192 | -0.179 | 0.232 | -0.267 |
| **Bio6** | **0.904** | 0.130 | -0.098 | -0.035 | 0.389 |
| **Bio7** | -0.070 | -0.407 | -0.095 | 0.333 | **-0.838** |
| **Bio8** | **0.922** | -0.228 | -0.046 | 0.260 | -0.007 |
| **Bio9** | **0.938** | 0.113 | -0.193 | -0.119 | 0.103 |
| **Bio10** | **0.937** | -0.127 | -0.128 | 0.297 | -0.005 |
| **Bio11** | **0.972** | 0.002 | -0.143 | -0.138 | 0.115 |
| **Bio12** | -0.199 | 0.501 | **0.822** | -0.087 | 0.106 |
| **Bio13** | -0.308 | 0.180 | **0.858** | -0.010 | 0.169 |
| **Bio14** | -0.077 | **0.928** | 0.221 | -0.140 | 0.188 |
| **Bio15** | 0.022 | **-0.920** | 0.162 | 0.243 | -0.116 |
| **Bio16** | -0.188 | 0.145 | **0.952** | 0.013 | 0.106 |
| **Bio17** | -0.087 | **0.927** | 0.262 | -0.104 | 0.198 |
| **Bio18** | -0.089 | -0.101 | **0.912** | 0.102 | -0.102 |
| **Bio19** | -0.049 | **0.940** | 0.164 | -0.100 | 0.160 |
| **Explained variation** | 6.402 | 4.236 | 3.480 | 2.264 | 2.060 |
| **Proportion of total variance** | **0.337** | **0.223** | **0.183** | **0.112** | **0.108** |

**TableS2.** Results of PCA for the bioclimatic variables of the Greater Antilles (in varimax-raw rotated coordinate system) by eigenvalues.

|  | **Eigenvalue** | **% Total variance** | **Cumulative eigenvalue** | **Cumulative %** |
| --- | --- | --- | --- | --- |
| **BioPC1** | 7.791 | 41.006 | 7.791 | 41.006 |
| **BioPC2** | 5.115 | 26.923 | 12.906 | 67.929 |
| **BioPC3** | 2.906 | 15.294 | 15.812 | 83.222 |
| **BioPC4** | 1.463 | 7.700 | 17.275 | 90.922 |
| **BioPC5** | 1.166 | 6.139 | 18.441 | 97.061 |

**Table S3**. Differences between Jamaica and other islands according to Kruskal-Wallis ANOVA. Largest differences: largest KW-H; analysis was done on dataset of 100 random point values for Hispaniola, Cuba and Puerto Rico, and all data points for Jamaica.

|  | **KW-H** | **P value** |
| --- | --- | --- |
| **BioPC1** | 2.066 | 0.1506 |
| **BioPC2** | 4.398 | 0.0360 |
| **BioPC3** | 56.221 | <0.0001 |
| **BioPC4** | 72.362 | <0.0001 |
| **BioPC5** | 233.572 | <0.0001 |

**Table S4.** Complete results table of AICc based model selection for ECC. Models are: 1 - Global model (Elevation + BioPC1-5), 2 - Elevation only, 3 - All BioPCs – Elevation, 4 – Elevation-correlated BioPCs, 5 Elevation-independent BioPCs, 6 -10 Each BioPC separately.

| **Hispaniola** | **K** | **AICc** | | **Delta AICc** | | **AICcWt** | **Cum.Wt** | **LL** | |
| --- | --- | --- | --- | --- | --- | --- | --- | --- | --- |
| Model 1 | 8 | 4617.10 | | 0.00 | | 1 | 1 | -2300.53 | |
| Model 3 | 7 | 4684.08 | | 66.98 | | 0 | 1 | -2335.02 | |
| Model 4 | 4 | 5489.77 | | 872.67 | | 0 | 1 | -2740.88 | |
| Model 2 | 3 | 5661.35 | | 1044.25 | | 0 | 1 | -2827.67 | |
| Model 5 | 6 | 5813.62 | | 1196.52 | | 0 | 1 | -2900.80 | |
| **Cuba** | **K** | **AICc** | | **Delta AICc** | | **AICcWt** | **Cum.Wt** | **LL** | |
| Model 1 | 8 | 3452.47 | | 0.00 | | 1 | 1 | -1718.22 | |
| Model 3 | 7 | 4129.38 | | 676.92 | | 0 | 1 | -2057.68 | |
| Model 5 | 5 | 7553.82 | | 4101.36 | | 0 | 1 | -3771.91 | |
| Model 4 | 5 | 7441.70 | | 3989.23 | | 0 | 1 | -3715.84 | |
| Model 2 | 3 | 8915.91 | | 5463.44 | | 0 | 1 | -4454.95 | |
| **Puerto Rico** | **K** | **AICc** | **Delta AICc** | | **AICcWt** | | **Cum.Wt** | **LL** | |
| Model 1 | 8 | -33.42 | 0.00 | | 0.89 | | 0.89 | 24.88 | |
| Model 3 | 7 | -29.23 | 4.18 | | 0.11 | | 1.00 | 21.75 | |
| Model 4 | 6 | -13.34 | 20.08 | | 0.00 | | 1.00 | 12.77 | |
| Model 2 | 3 | 79.35 | 112.77 | | 0.00 | | 1.00 | -36.65 | |
| Model 5 | 4 | 289.06 | 322.48 | | 0.00 | | 1.00 | -140.48 | |
| **Jamaica** | **K** | **AICc** | **Delta AICc** | | **AICcWt** | | **Cum.Wt** | | **LL** |
| Model 3 | 7 | 63.8430 | 0.0000 | | 0.6664 | | 0.6664 | | -24.8162 |
| Model 1 | 8 | 65.2264 | 1.3835 | | 0.3336 | | 1.0000 | | -24.4776 |
| Model 7 | 3 | 111.1842 | 47.3412 | | 0.0000 | | 1.0000 | | -52.5697 |
| Model 8 | 3 | 140.6150 | 76.7721 | | 0.0000 | | 1.0000 | | -67.2851 |
| Model 10 | 3 | 154.4886 | 90.6456 | | 0.0000 | | 1.0000 | | -74.2219 |
| Model 9 | 3 | 158.2249 | 94.3819 | | 0.0000 | | 1.0000 | | -76.0900 |
| Model 2 | 3 | 167.9651 | 104.1222 | | 0.0000 | | 1.0000 | | -80.9602 |
| Model 6 | 3 | 169.8889 | 106.0460 | | 0.0000 | | 1.0000 | | -81.9221 |
